# Plasma–liquid interactions in the presence of organic matter—A perspective

**DOI:** 10.1063/5.0203125

**Published:** 2024-04-26

**Authors:** Katharina Stapelmann, Sophia Gershman, Vandana Miller

**Affiliations:** 1 Department of Nuclear Engineering, North Carolina State University, Raleigh, North Carolina 27695, USA; 2 Princeton Plasma Physics Laboratory, Princeton, New Jersey 08540, USA; 3 Center for Molecular Virology and Gene Therapy, Institute for Molecular Medicine and Infectious Disease, Department of Microbiology and Immunology, Drexel University College of Medicine, Philadelphia, Pennsylvania 19129, USA

## Abstract

As investigations in the biomedical applications of plasma advance, a demand for describing safe and efficacious delivery of plasma is emerging. It is quite clear that not all plasmas are “equal” for all applications. This Perspective discusses limitations of the existing parameters used to define plasma in context of the need for the “right plasma” at the “right dose” for each “disease system.” The validity of results extrapolated from *in vitro* studies to preclinical and clinical applications is discussed. We make a case for studying the whole system as a single unit, *in situ*. Furthermore, we argue that while plasma-generated chemical species are the proposed key effectors in biological systems, the contribution of physical effectors (electric fields, surface charging, dielectric properties of target, changes in gap electric fields, etc.) must not be ignored.

## I. INTRODUCTION

Non-equilibrium plasma in contact with a liquid state is of relevance for a broad range of applications including but not limited to material science, environmental remediation, agriculture, and for plasma medicine.^1^ Applications of plasma for medical purposes involve the presence of a liquid state that is often comprised of multiple components, each contributing to plasma–liquid interactions.^2^ This Perspective will discuss these interactions as relevant for plasma sources where the active plasma is in direct contact with the interface; the system then consists of plasma, the plasma–liquid interface, and the bulk liquid containing inorganic molecules and the organic matter (defined generically as a source of carbon-based compounds including carbohydrates, lipids, proteins, and nucleic acids) specific for the application of interest. The simplified flow of events begins with reactive species produced in the plasma, which are transported into the liquid and react with substances in the liquid. The organic matter in liquids then becomes part of the chemistry. It can be a passive target for reactions, changing the constitution of the cocktail of reactive species or actively changing the chemistry through enzymic or antioxidant reactions, as seen when living cells are present. Changing chemistry in the liquid phase, in turn, impacts the equilibrium densities, thus, mass transfer through the plasma–liquid interface, and, consequently, the densities of species in the gas phase.^3^

In addition to the chemical interactions between the plasma and target, there is an electrical connection between the plasma source and target. The power delivered to the plasma depends on the overall circuit impedance, which, in turn, is affected by the electrical properties of the target. Therefore, the target can impact the power deposited in the plasma that then impacts plasma parameters and plasma chemistry in the gas phase.^4^ This changes the equilibrium densities as well as transport of species across the plasma–liquid interface.^3^ Changes in the target material, e.g., conductivity or permittivity of the material, alter the electrical properties of the target. While these changes may be initiated by plasma-induced chemistry, cells also contribute to changes in the electric circuit, when present.^5^ As cells respond to plasma exposure, their electrical properties and the properties of the cell-containing target change, and so does their contribution to the electric circuit. Moreover, changing the dielectric properties of the target impacts surface charging and sheath formation,^6^ directly affecting the plasma as the sheath at the liquid surface determines the boundary conditions of the plasma.^7^ The deposited power in the plasma discharge, the properties of the target, here a liquid with the presence of organic matter, and the presence of impurities all influence the electric field and the (bidirectional) mass and energy transfer across the plasma–liquid interface.

**FIG. 1.**
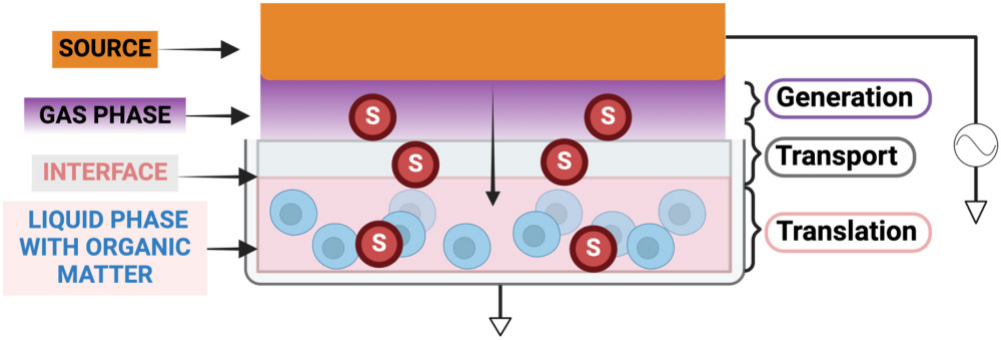
When cells are exposed to plasma, the deposited power in the plasma discharge, the properties of the target, here a liquid with the presence of organic matter, and the presence of impurities influence the electric field and the (bidirectional) mass and energy transfer across the plasma–liquid interface. They act as a single system interacting chemically and electrically.

This truly interactive behavior between plasma and biological systems occurs simultaneously at two interconnected levels: chemically and electrically. In this Perspective, we aim to advocate for the analysis of the system as a whole—plasma source, gas phase plasma, liquid phase and the organic matter—and to raise the level of awareness regarding the importance of this complex system that interacts at multiple levels. We describe the generation and transport of reactive species, in particular, reactive oxygen and nitrogen species (RONS) from the gas phase into the liquid phase (Sec. II) with an emphasis on plasma–liquid interactions in the context of plasma medicine. In Sec. III, the complexity of the system is increased by introducing organic matter in the liquid. We argue why the system needs to be studied as “one system” and motivate more research toward transport processes and interface chemistry (Fig. 1). Sections IV and V introduce cells to the system, with an emphasis on the chemical and biological implications in Sec. IV and on the electric circuit and feedback to the whole system in Sec. V. Finally, Sec. VI concludes the Perspective with a summary of the new insights and recommendations for future research.

## II. GENERATION AND TRANSPORT OF REACTIVE SPECIES

### A. The gas phase

Reactive species are produced in plasmas by dissociation and the formation of molecules in the plasma or the plasma afterglow. Depending on the process gas and the plasma device used, the cocktail of reactive species differs; in general, in the presence of air with humidity, the most common reactive species produced are O_3_, OH and H_2_O_2_, NO and N_x_O_y_. If the plasma or the effluent of a plasma is in contact with a liquid, reactive species will be transported from the gas phase through the gas–liquid interface into the bulk liquid. Different setups can be considered for gas–liquid transport, among them direct discharges in liquids, gas phase plasmas producing reactivity in the liquid, and multiphase plasmas including gas phase plasmas with liquid droplets or aerosols and gas bubbles in liquids.^1^ For the purpose of this Perspective, the focus shall be on gas phase plasmas producing reactivity in the liquid. A comprehensive review of this type of plasma–liquid interaction, as well as of the other types listed above, can be found in Ref. 1.

There are two commonly used approaches to produce reactivity in liquid by gas phase plasmas, dielectric barrier discharges (DBD) and plasma jets. Here, it is useful to distinguish between direct contact plasma and indirect contact plasma: we describe a discharge as direct contact plasma when the active plasma region is in contact with the treated object. Examples include a DBD where the treated object becomes the counter electrode or an ionization wave jet, also known as DBD-jets, when the ionization wave is touching the target. In this case, the target is exposed to the electric field, charged species, metastables, and becomes a part of the electric circuit. Indirect contact plasma, on the other hand, describes plasma sources where the target is only in contact with the effluent; the active plasma is not in direct contact with the target, and the target is only exposed to the long-lived species and, depending on the setup, UV radiation. Examples for these setups include surface DBDs^8–11^ and the COST jet.^12–14^ The discussion in this Perspective is limited to direct contact plasma to analyze the interactions and feedback between the plasma and the treated object for biologically relevant systems.

### B. Plasma–liquid interactions

Plasma–liquid interactions have been a subject of research interest for centuries, with shifting focuses and applications. Starting with the production of nitric acid by an electric spark in air in the late 1700s,^15^ to electrolysis in the 1800s,^16^ followed by the investigation of electric breakdowns in dielectric liquids in the 1900s,^17^ more recently, the focus has been on environmental and medical applications.^18^ For a comprehensive introduction to the field of plasma–liquid interactions, the reader is referred to Bruggeman *et al*.'s “Plasma-Liquid Interactions: Review and Roadmap”^1^ and the topical review on recent progresses and challenges in Ref. 19. We provide a brief outlook here limited to issues relevant to plasma medicine.

An important and under-researched aspect of plasma–liquid interactions is the plasma sheath when direct plasma devices are used, as recently pointed out by Vanraes and Bogaerts.^7^ The plasma sheath at a liquid surface determines the boundary conditions of the plasma phase and impacts (bidirectional) mass and energy transport across the boundary. Furthermore, the boundary conditions for Maxwell's equations are determined by the sheath, affecting the behavior of plasma and liquid due to their electric coupling. For more details, the reader is referred to their Perspective paper.^7^ Chemistry in the plasma sheath may differ significantly from the chemistry in the bulk plasma due to higher local electric fields and different charged particle densities and energy distributions. In addition to the plasma sheath at the plasma–liquid boundary, the interface itself can lead to unique chemistry, as discussed in Sec. II C.

### C. The interface

While much of the fundamental understanding of chemistry originates in bulk homogeneous phases, many reactions of interest occur at interfaces.^20^ The air–liquid interface extends from 0.3 to 0.6 nm under ambient conditions.^1^ While the air–liquid interface is easy to define macroscopically, it is more challenging at nanoscale. Thermal fluctuations lead to fast morphological changes over short time and length scales, which makes it difficult to define the interface precisely.^20^ Generally, the interface is distinguished by a density gradient from the denser liquid medium to the much less dense air or gas phase. Molecules and atoms in this region are not in a well-defined phase.^20^ Water molecules, for example, tend to orient with their hydrogen atoms toward the gas phase; at 0.5–1 nm distance, the water molecules are once again randomly oriented.^21^ In the presence of dissolved salts in the water, the concentration of ions may vary over a larger distance, characterized by the Debye length.^21^ The gradients in concentration and density are associated with gradients in permittivity, conductivity, etc., on small length scales (sub-nanometer to a few nanometer). The impact of these gradient changes on transport of species and the plasma in contact with the interface is an unexplored topic and warrants more attention. As gas–liquid interfaces (without plasma) themselves are still a topic of active research, collaborations with surface and interface science would facilitate the exploration of the phenomena together. Infrared and sum frequency generation spectroscopy have been employed in recent years to study molecules at fluid interfaces.^21^ Laser absorption in a total internal reflection setup has been used to probe interface-near solvated electrons,^22,23^ and the emergence of femtosecond lasers and fast imaging allows probing of interface-near species using two-photon absorption laser-induced fluorescence.^24^ Nonetheless, these diagnostic methods have not been widely employed yet and are not readily available. In addition, probing interfaces with optical diagnostics creates challenges for data interpretation and calibration, as vignetting, reflections, and potentially moving interfaces (e.g., evaporation, oscillations, and dimple dynamics) impact the results.^25,26^

### D. Transport

**FIG. 2.**
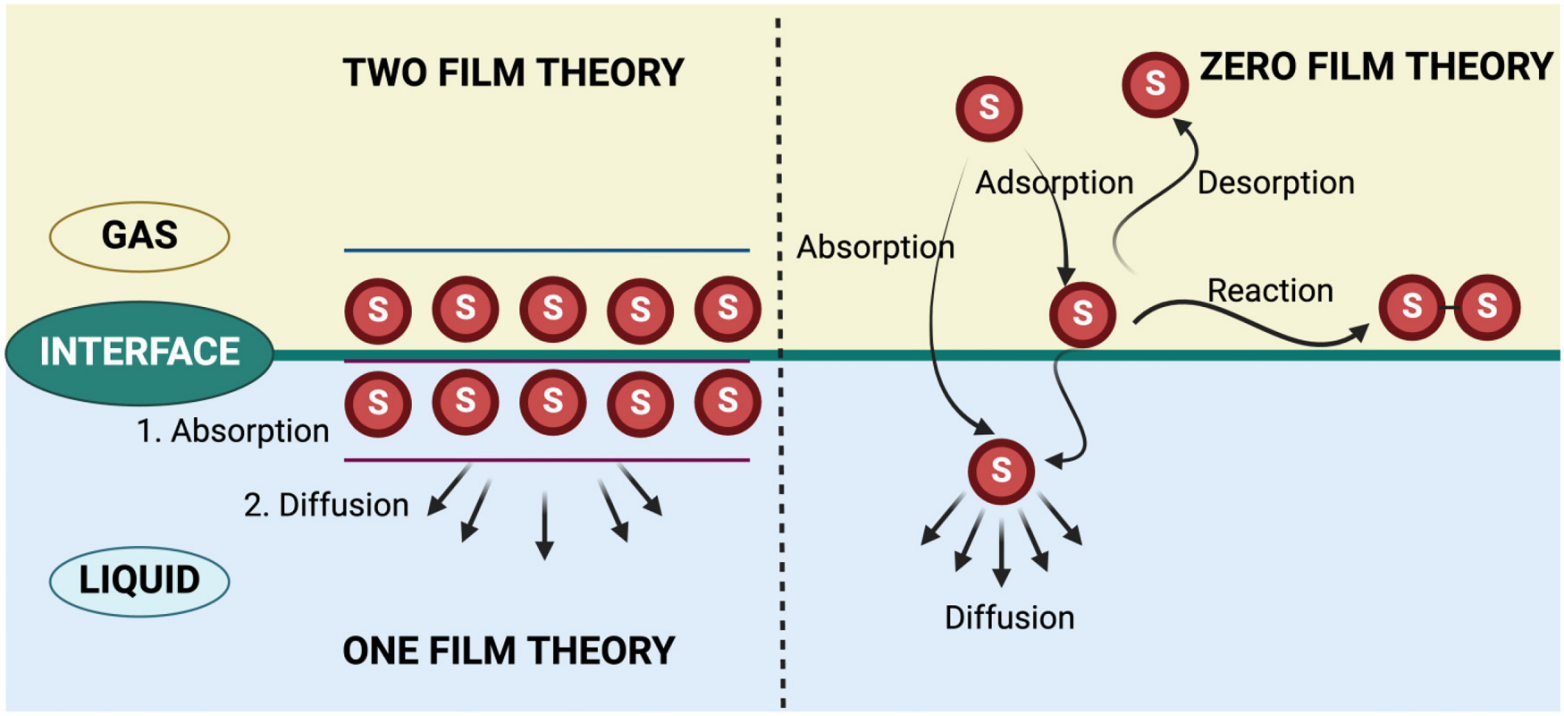
Transport across a gas–liquid interface. Neutral species (S) can be absorbed or adsorbed at the surface. Adsorbed species can react, desorb, and return into the gas phase or absorb and diffuse into the bulk liquid. According to the zero-film theory, there is no resistance for the flux. One-film and two-film theory add resistance to the flux of neutral species by introducing a stagnant liquid layer and two stagnant layers on each side, respectively.

Once the species approach the interface, the uptake of gases is a complex interaction that is governed by gas- and condensed phase parameters and the properties of the interface. In a general gas–liquid interface, neutral species diffuse through the gas phase to the liquid surface (Fig. 2). At the surface, species can be absorbed (dissolve in the liquid), adsorb (adhering to the surface) and react, or desorb and return into the gas phase. Adsorbed species can also solvate into the liquid and penetrate the bulk liquid, followed by diffusion in the bulk liquid.^27^ The transport of species into the liquid has traditionally been described by the zero-film interfacial transport theory,^28^ where there is no resistance to the flux of gaseous species into the liquid and the concentration equilibrates instantaneously in both phases. Henry's law is an example.^29^ Albeit commonly used, the applicability and correctness of Henry's law is questionable for non-equilibrium plasmas in contact with liquids. First, Henry's law is based on equilibrium concentrations on each side of the interface, which is not always the case in non-equilibrium and transient atmospheric pressure plasmas. Second, Henry's law assumes free diffusion. In addition to diffusion, transport through the plasma–liquid interface can be influenced by electrohydrodynamic forces, electric surface stresses, and even more forces in the case of plasma jets with a gas flow.^30,31^

Transport is studied more generally using the one-film theory, where a stagnant liquid film is introduced at the interface. The stagnant liquid film serves to absorb species before they diffuse into the bulk liquid. In other words, the element of resistance to the flux of gaseous species is introduced. Species accumulate in the film until equilibrium with the gas phase is reached, preventing the uptake of more species until the concentration in the film is reduced by diffusion into the bulk liquid.^28^ The two-film theory, widely used in environmental and aerosol science, provides further improvements by adding a second stagnant film at the interface. The presence of the second film on the gas side of the interface limits the transport of hydrophilic species, slowing their uptake into the liquid phase. Hydrophobic species on the aqueous side experience a similar slowing of desorption from the liquid phase.^28^

Recent work by Silsby *et al*. has found the two-film theory superior in modeling the mass transfer across a gas–liquid interface.^28^ A drawback of the two-film theory is the requirement of a Sherwood number, a dimensionless number representing the ratio of convective mass transfer to diffusive mass transport. The authors state that “due to the high reactivity of most plasma generated species, there is very little experimentally validated data.”^28^ In addition to the lack of experimentally validated data, the complexity of the models and computational cost increase significantly when spatial dependences need to be resolved, i.e., if 1D or 2D models are applied. For transport across the air–water interface, global models (0D) are often used where each region is solved separately and spatially averaged to reduce computing cost. The two-film theory can be implemented via flux equations across the gas–liquid interface by introducing a resistance factor, as described in Ref. 28,

$$\Gamma_{i,\mathrm{gl}}=\left(C_{i,g}-\frac{C_{i,l}}{H_{i}^{CC}}\right)\cdot \frac{1}{R_{i}},$$

with $\Gamma_{i,\mathrm{gl}}$ being the flux of the *i*th species across the gas–liquid interface, $H_{i}^{CC}$ being the dimensionless Henry's law constant of the *i*th species, $C_{i,g}$ being the concentration of the *i*th species in the gas (*l*, liquid) phase, and $R_{i}$ being the resistance to mass transfer caused by the interface.^28,32^

Although the implementation of a two-film theory can improve the examination of transport in models, substantial research is needed to understand the interfacial transport in non-equilibrium conditions and not just on the plasma side. Evaporation, as a non-equilibrium condition, is still not well understood and captured on the interface's short time and length scales.^20^ Including the non-equilibrium conditions of the plasma, combined with the chemical reactivity and the influence of electric fields, current, electrohydrodynamic forces, and a plasma sheath, complicates the description of the processes on the interface's length and time scales, which span orders of magnitudes depending on the phase and the species in question. Limitations in diagnostics to capture phenomena on nanometer and sub-nanometer length scales and picosecond time scales make these experiments especially challenging. One approach to overcome this limitation could be a combination of experiments where phenomena on much larger time and length scales are experimentally captured and coupled with improved models predicting the experimental scale outcomes. These may help better understand the interfacial transport and the impact of the non-equilibrium plasma on the interface. Particularly interesting is the behavior of the constituents in the liquid. Surfactants, for example, adsorb at fluid interfaces and can be directly exposed to the plasma at the plasma–liquid interface. Other components may be dissolved in the bulk liquid or in the case of adherent cells at the bottom of the well. Nonetheless, as the targets start to react with plasma-induced chemistry, the equilibrium densities in the gas and the liquid phase change, impacting the transport across the interface bidirectionally. Resolving interface processes in a plasma–liquid setup relevant for plasma medicine applications, e.g., while exposing cells in culture, poses an enormous experimental challenge. However, deeper understanding of transport across interfaces with non-equilibrium conditions would be of interest to a broader scientific community.

### E. Liquid phase

Once the species enter the liquid phase, the next factor that determines species abundance is their lifetime and penetration depth into the liquid. OH, for example, was shown to penetrate on the order of 100 nm in neat water where the predominant loss mechanism is the formation of H_2_O_2_.^23^ Similarly, an average penetration depth for electrons was determined to be 10−100 nm.^23^ NO as one of the biologically relevant RNS is a relatively stable radical but with low water solubility. Verlackt *et al*. show computationally that NO is only present at the interface.^33^ However, species do not just penetrate from the gas phase into the liquid phase. Reactions in the interface and the bulk liquid can produce short-lived reactive oxygen species (ROS) and RNS. As an example, NO may be created by the reactions: NO_2_ + O → NO + O_2_ and NO_2_ + O_3_ → NO + 2 O_2_, among others.^34^

Once inside the bulk liquid, species diffuse depending on their lifetime and penetration depth. In addition to regular diffusion, electrohydrodynamic induced flow and recirculation as well as electric surface stresses across the interface induced flow may influence the distribution of species, depending on the plasma device used.^31,35,36^ In the case of jet plasmas with a gas flow, a deformation of the plasma–liquid interface and flow inside the bulk liquid created by the gas jet momentum, rotational flow pressure, tangential shear stress, surface tension, and buoyancy are added to the system.^37^ The gas flow and associated forces, thus, have additional effects on the distribution of species in the liquid: depending on the gas flow, hydrodynamic instabilities can occur leading to oscillating dimple dynamics enhancing convective effects and increasing the complexity of the system.^25^

Plasma behavior and transport processes inside the liquid system can also depend on the type of liquid used. Dickenson *et al*. demonstrated that the dominant mechanism driving a liquid flow changes with the charge relaxation time of the liquid.^31^ If the charge relaxation time of the liquid is longer than the characteristic time of the plasma, the liquid behaves like a dielectric and the flow is mainly induced by electric surface stresses. On the other hand, when the charge relaxation time is shorter than the characteristic plasma time, the liquid behaves as a conductor and the flow is dominated by electrohydrodynamic forces.^31^ This has great implications for all applications working with liquids. Tap water has a relaxation time of approximately tens of nanoseconds, whereas the relaxation time of the cell culture medium is less than 1 ns. In de-ionized water (relaxation time of 1–10 μs), the charged species accumulate on the dielectric water surface for longer times, reducing the electric field on the gas side of the interface and increasing the electric field on the liquid side of the interface.^31^ When surfactants are present, the plasma-induced liquid flow was shown to depend strongly on the initial surfactant concentration.^38^ Kawasaki *et al*. observed a change in the flow direction and strength depending on the surfactant concentration. Surface tension was found to be altered during plasma treatment due to surfactant decomposition, and the Marangoni flow generated by the surface tension gradient was identified as the driving force.^38^

More research, computationally and experimentally, toward the impact and changes of the liquid properties during plasma treatment, including the flow behavior and, thus, the transport of species inside the liquid, would be desirable. For example, the use of fluorescent dyes and imaging, particle imaging velocimetry,^39^ or Schlieren imaging^35,36^ already provides a wealth of information regarding the transport of species in the liquid. Combined with computational efforts, it will help to explore the forces at work and how plasma interacts with and impacts the liquid.

This section discussed the state of knowledge of phenomena that influence the transport of species across the interface and the transport within the liquid. In the context of plasma medicine applications, the extent of impact of these factors on biological outcomes is unclear. Most studies focus on the plasma, the liquid, or the biological outcome, and only little is known about the whole system and the rapidly evolving interactions between plasma and liquid in the presence of cells and the interactions with the cells themselves. These limitations can only be addressed by more interdisciplinary and collaborative studies.

*Plasma–liquid interactions in the context of plasma medicine:*

Since the discovery that plasma can be safely applied to cells and living tissues, tremendous progress has been made in the use of plasma to controllably achieve diverse biological effects ranging from the stimulation of cell division and differentiation to the induction of different cell death pathways.^18,40^ Based on these cellular effects, multiple medical applications of plasma are being explored, including disinfection, treatment of skin diseases, wound healing, and more recently as a broad-spectrum anti-cancer tool.^41–43^ The association of liquids in these applications is unavoidable and has been explored somewhat. The importance of the influence of organic constituents in the solution has been recognized only recently and will be addressed in more detail in Sec. III.

## III. PLASMA–LIQUID INTERACTIONS IN THE PRESENCE OF ORGANIC MATTER

The recognition of the importance of organic constituents, buffering capacity of the solution, and contaminants in changing the chemistry in the plasma community is relatively recent. Experiments to investigate the origin of reactive species (gas vs liquid phase) with the reference microscale atmospheric pressure plasma COST jet (active plasma is not in direct contact with the liquid interface) show that reactive species originate exclusively in the gas phase when pure water is treated.^44,45^ On the other hand, when solutions containing cysteine (organic matter) were exposed to plasma, reactive species originating in the liquid phase become part of the reaction pathways.^46^ This strongly suggests that when organic matter is present in the liquid, the reaction products incorporate ROS originating in the liquid phase. It also highlights that the target can modify the chemistry.

Therefore, it is particularly important to investigate the whole plasma system for each specific application under investigation in a setting as realistic as possible. In agricultural applications, while experiments using de-ionized (DI) water may be suitable and useful for reproducibility under lab conditions, the researcher should be encouraged to use well water or tap water as well to translate results from the lab to the field. Similarly, the concentrations and evolution of long-lived species H_2_O_2_ and NO_2_^−^ produced by a DBD are different between DI water and cell culture medium,^13^ and even more so when cells are present.^47^ As discussed in a recent roadmap paper,^18^ the presence of organic matter that may become part of chemical reaction pathways needs to be studied in more detail to understand, optimize, and commercialize plasma devices for each application individually.

It is apparent from Sec. II that the transport of species from the gas phase to the liquid phase is rather complex, even if the liquid under investigation is just pure water. Chemical reactions occur in the gas and in the liquid, while reactions in and at the interface are accompanied by transport processes. A simplistic way to consider transport of species across the interface is to look at equilibrium conditions with Henry's law as one of the simple relationships describing the density distributions of species in the gas phase and the liquid dependent on their Henry's law constant. Considering now a liquid that contains organic material, i.e., a target for the reactive species to react with, the density of reactive species in the liquid declines because the reactive species are consumed by reactions with the target. For example, ozone has a low Henry's law constant, and thus, the transport of ozone into the liquid is inefficient and it enters the liquid in small amounts. If peptidoglycan is present in the liquid, ozone reacts with it and is consumed in the liquid phase, allowing ozone to enter the liquid again, depleting the gas phase of ozone. Lietz and Kushner^3^ have elegantly demonstrated this effect computationally. Their study highlights that organic matter in liquid not only influences the liquid phase chemistry but also the gas phase chemistry by changing the efficiency by which different species enter the liquid phase.

Furthermore, different chemical groups impact the chemistry differently, and different reactive species originating in the plasma will modify targets differently. For example, when atomic oxygen is generated in the presence of ring structures, O enters the liquid and upon hydrogen abstraction from the C–H bonds, forms hydrogen peroxide.^48,49^ As a result, the hydrogen peroxide concentration in the liquid can increase significantly. Cysteine, a thiol (-SH) containing amino acid often used as a simple model to detect chemical modifications,^50,51^ consumes or converts hydrogen peroxide, leading to significantly lower hydrogen peroxide concentrations in the liquid.^46^ Interestingly, hydrogen peroxide concentration does not change when cysteine is mixed with commercially available hydrogen peroxide at the same concentration produced by plasma. An initial reaction with short-lived species from the plasma is necessary to facilitate the reactions of hydrogen peroxide with the cysteine derivatives that may be transient and non-stable in nature.^51^

Proceeding from a single organic component in the liquid to cell culture medium, a mix of amino acids, vitamins, inorganic salts, glucose, and antioxidants, supplemented with serum and penicillin/streptomycin, increases the complexity of the chemical reactions that can occur. In Ranieri *et al*.,^13^ we reported a significant increase in hydrogen peroxide concentration when the cell culture medium was exposed to plasma compared to plasma exposure of water. Similarly, nitrite concentrations were higher in cell culture medium exposed to plasma. This exemplarily highlights the changes reactive species undergo upon entering the liquid. The chemical components in the liquid offer reaction targets for plasma-generated species, and the cocktail of reactive species is further modified by these secondary interactions with the components of the cell culture medium. This implies that some of the reactivity may be transferred to the modified chemical groups, which could potentially lead to “translation” of plasma-generated RONS to less reactive but stable, more bioactive molecules. A limited number of laboratories have investigated this very important aspect that has huge potential to facilitate the therapeutic use of plasma. Plasma-treated physiological cysteine solution (2 mM) did not affect the migration and proliferation of human keratinocytes; at higher, albeit non-physiological cysteine concentrations (100 mM), a reduction in migration and proliferation, accompanied by a decreased intracellular ROS level and increased pro-inflammatory cytokine secretion, was observed.^52^ The use of plasma-treated n-acetylcysteine,^53–55^ leucine,^56^ phenylalanine, and tyrosine^57^ solutions has been investigated as effective antimicrobial agents; methionine and tryptophan solutions have demonstrated antitumor effects.^58^ There is a dire need for research toward the understanding of the physiological impact of plasma-generated RONS directly, their translation into more stable bioactive molecules, and the effect on living organisms and their interplay with RONS.

## IV. CELL RESPONSE TO RONS

**FIG. 3.**
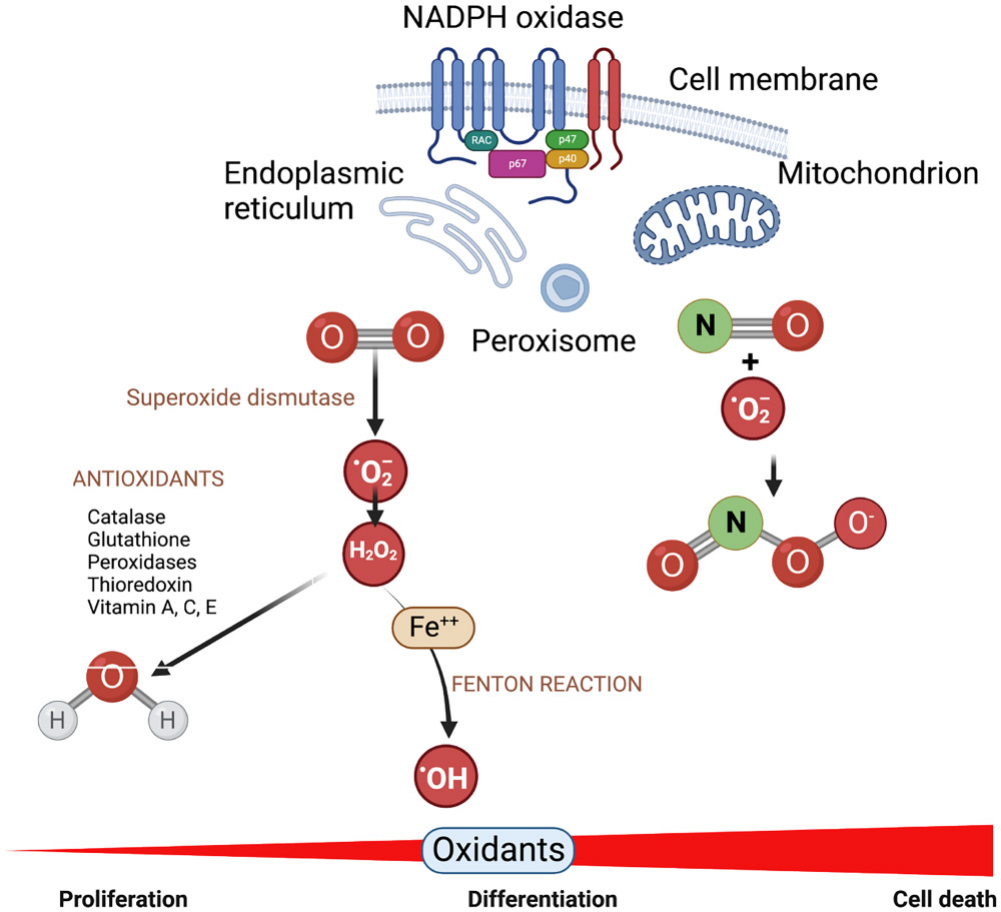
Cell redox balance. Enzymatic and non-enzymatic reactions generate superoxide anion radical and hydrogen peroxide in different cell organelle. In the presence of reduced transition metals, hydrogen peroxide is converted to highly reactive hydroxyl radical. Superoxide can also combine non-enzymically with nitric oxide to produce peroxynitrite that can oxidize or nitrosylate some amino acids including cysteine, tyrosine, methionine, and tryptophan. When cell redox sensor systems detect deviation from the steady state redox for that cell, a system of antioxidants functions to restore the balance. The level of oxidants defines the impact on cell outcome.

Redox or oxidation and reduction processes are essential for normal function of living cells.^59^ Cells produce RONS, including superoxide (O_2_^−^), hydroxyl radical (HO**.**), hydrogen peroxide (H_2_O_2_), nitric oxide (NO**.**), and peroxynitrite (ONOO_−_), as by-products of cellular metabolism see Figure 3.^60^ Cellular enzymes convert the relatively inert oxygen to reactive radicals that can oxidize lipids and proteins in cells, hence the name “reactive oxygen species (ROS).”^61^ Reactive oxygen species, from the perspective of a cell, encompass different oxidant molecules that affect many biological functions based on their molecular properties.^62^ Whether produced intracellularly through normal physiological functions, delivered exogenously, or be triggered by extraneous sources, RONS alter the redox status of the cells by a process often referred to as oxidative stress.^63^ The cellular outcomes of this oxidative stress include signaling, adaptation, repair, or death and are determined by the concentration of RONS (including relative concentrations of different species), the status of the antioxidants in the cell, the kind of cell and in a complex tissue, and the neighboring cells.^64^ As applicable to plasma medicine, this means future studies must focus on each “plasma-disease-tissue” system individually.

Low levels of ROS are harmless and often function as signaling or regulatory molecules for many cellular pathways that are necessary for cell survival. They also help maintain the function of other redox-sensitive signaling proteins.^61^ They are essential for maintaining physiological homeostasis. Cells are also able to recover from small, transient exposure to external RONS using physiological responses.^60^ Higher concentrations of ROS, whether produced by the cell itself or from external sources, can be detrimental for cell survival and function. Disturbances to cell redox balance are restored by controlled destruction or scavenging of RONS through an extensive system of antioxidant machinery.^59^ Antioxidants protect tissues by either promoting adaptation to RONS or by triggering cell death pathways if the cells cannot be rescued. An assessment of the antioxidant capacity of the cells then becomes a vital part of efficacy studies in different disease models.

Once the plasma generated primary RONS and the secondary species reach the cell, they have two possible fates: they may interact with molecules on the cell membrane or diffuse through the cell membrane freely and enter the cell. The outermost layer of the cell may provide another target for chemical species to react with, forming different species and changing the chemistry cocktail “passively.” Many of the RONS present in plasma are not unknown to cells and, therefore, become important players in the regulation of cell and tissue homeostasis by challenging the redox balance.^43^ The topic of plasma-generated RONS in the context of redox biology has been extensively discussed in Refs. 40 and 65.

Exposure to plasma-generated (exogenous) RONS may also elicit the formation of intracellular RONS.^61^ The cell must determine whether these exogenous oxidants are physiological and tailor its response accordingly. Since the use of plasma is transient, this Perspective only examines the effect of acute RONS challenge faced by cells.

### A. Interactions between plasma RONS and cell membrane

The cell membrane, as the first interaction target for RONS, is a semipermeable lipid bilayer that serves as a barrier between the interior of a cell and the external environment. Through its biomechanical properties, it maintains cell constituents at a steady level by regulating the transport of nutrients into the cell and byproducts of cell metabolism out of the cell. It also has many proteins embedded in it that are necessary for signaling as well as some that act as transport channels.

**FIG. 4.**
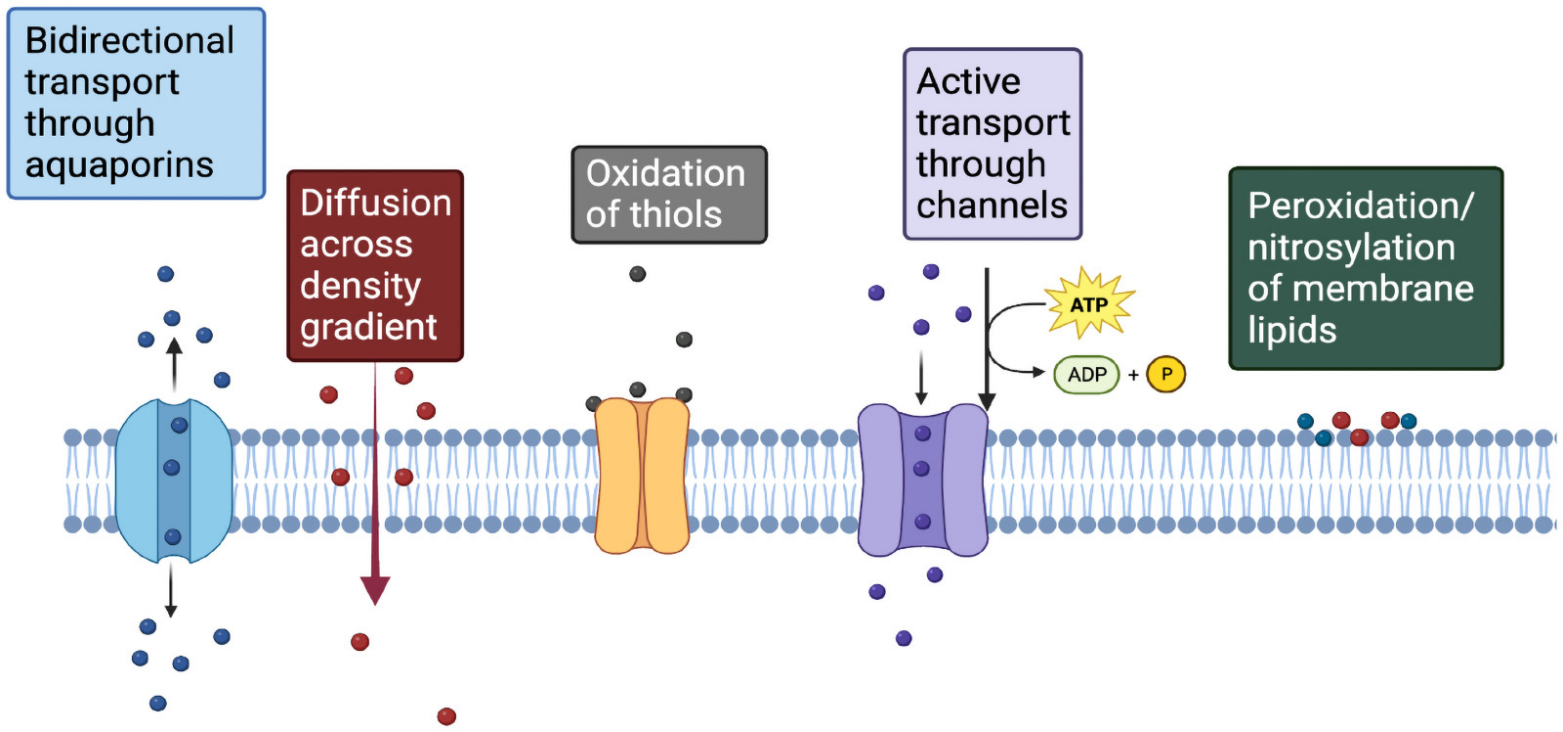
The possible fate of plasma RONS when they reach the cell membrane.

When plasma RONS reach the outside of a cell membrane, they interact with different molecular components of the cell membrane (Fig. 4), resulting in diverse outcomes. These include bidirectional transport through aquaporins, active transport through channels, diffusion across a density gradient, peroxidation/nitrosylation of membrane lipids, and the oxidation of thiols (Fig. 4). These are described in more detail below using specific species as examples.

Transport: Superoxide cannot diffuse across cell membranes at physiologic pH, except through voltage-dependent ion channels,^66^ and its reported half-life is less than a couple of seconds.^67^ It is rapidly converted to hydrogen peroxide by superoxide dismutase. This section will focus on how the cell membrane interacts with hydrogen peroxide, the most stable non-radical specie that is produced in plasma and solvates readily in aqueous fluids. Its biological properties depend on its chemical reactivity, half-life, and lipid solubility. Physiologically, it is a major redox signaling molecule in the cell, present in low nanomolar concentrations inside the cell.^68^ The extracellular compartments levels of H_2_O_2_ are reported to range from 1 to 5 *μ*mol.^69^ The specificity of signaling via H_2_O_2_ is controlled by its differential production and transport across subcellular compartments of the cell^70^ and relies on the oxidation of cysteine residues of proteins.^70^ Reversible oxidation of cysteines may be achieved at approximately 100 nM of H_2_O_2_, and because of its stability, these effects may be produced at distant sites, as much as 1600 *μ*m away.^71,72^

When plasma-produced H_2_O_2_ reaches the cell, it is assumed that it freely diffuses across the membrane across a concentration gradient. The permeability coefficient of H_2_O_2_ across the cell membrane of Jurkat T cells was measured as 2 × 10^−4^ cm s^−1^, and it took 0.9 s to establish a stable gradient.^73^ While it can cross lipid membranes, the diffusion efficiency is dependent on the lipid composition of the membrane and is compromised by the hydrophilic nature of H_2_O_2_.^73^ The length of cell surface fatty acids and their saturation, phosphorylation, and glycosylation influence the permeability of molecules across membranes.^74^ However, this cannot be the dominant method of regulating H_2_O_2_ transport in response to plasma RONS because it is a slow process and may only be useful for long-term adaptation strategies. Rapid cell responses, as seen immediately after plasma exposure, are likely to rely on specific aquaporins for transport of H_2_O_2_ across hydrophobic membranes.^75^ Recent reports also suggest that mechanical changes in cells as a consequence of changes in osmotic pressure can influence H_2_O_2_ diffusion across membranes.^76^

Peroxynitrite, another potent oxidant, is also known to pass through cell membranes with relative ease with calculated permeability of 8 × 10^−4^ s^−1^ (Ref. 77) and may diffuse up to 60 *μ*m.^72^

Peroxidation/Nitrosylation of lipids: Lipids in the cell membrane are a preferred target of RONS resulting in their peroxidation. The carbon–carbon double bonds in polyunsaturated lipids are particularly susceptible to the action of plasma oxidants such as O_2_^−^, HO**.**, and ONOO potentially changing their structure and function. Hydroxyl radicals are highly reactive and modify molecules within a few nanometers of their generation. Superoxide must be protonated to form hydroperoxyl radical (HO**.**_2_) that gets converted to hydrogen peroxide, which, in turn, becomes the source of more HO**^.^** to initiate lipid peroxidation.^78^ Low levels of lipid peroxidation induce cell survival pathways in cells through stress response mechanisms. When the oxidative damage to lipids overwhelms the cell's capacity to repair, the cell is pushed toward programmed death.^79^ Similarly, nitrosylative modifications of lipids changes cell signaling mechanisms that determine cell fate.^80^ These indirect signaling effects of RONS are outside the scope of this Perspective.

Membrane fluidity: The abundant lipid content of cell membranes imparts a highly fluid biophysical state and is a major determinant of fundamental cell functions like adhesion and migration. It also affects permeability, transport of molecules, osmotic stability, and enzymic functions. The more fluid a membrane, the more permeable. Peroxidation of lipids by exogenously added H_2_O_2_ decreases membrane fluidity, compromising protein function and interactions by increasing the numbers of lipid rafts.^81^ Computational studies indicate that this plasma RONS-mediated loss of fluidity may be transient and is followed by a sustained increase in fluidity.^82^

As translational applications for treatment of different diseases of plasma broaden, definition of safe plasma dose for each application will become an imperative that cannot be avoided. Investigations that help define the biochemical changes in cell membrane specific to interactions with plasma RONS (and other effectors in plasma) will facilitate this crucial need.

### B. Fate of plasma RONS inside the cell

The redox status of a cell is tightly regulated and any change in cell RONS is controlled by multiple redundant pathways, usually specific for each subcellular compartment.^70^ This is evident in the example of macrophages. Physiological amounts of RONS produced in the mitochondria serve largely as signaling molecules to regulate cell function, including innate immune responses.^83^ The large quantities of RONS produced as a result of an oxidative burst in phagolysosomes are fundamental for killing invading pathogens.^83^ However, the danger to other cell organelle is minimal because these toxic molecules are sequestered and rapidly destroyed or neutralized by the antioxidants resident in the phagolysosome.^84^ Small amounts of RONS are also released into the extracellular space, presumably to initiate signaling events to stimulate immune responses.

Once plasma RONS (primary or secondary species) show up inside the cell, their fate depends on the cell type, location in the subcellular compartments, their concentration, the specific RONS species, and the lifetime of those species.^70^ The assumption that they are equally distributed throughout the cell is incorrect. The robust antioxidant system of the cell probably neutralizes most of the species rapidly. However, long lived species at physiological concentrations may continue to interact with biomacromolecules to initiate specific physiological pathways that affect developmental and immunological processes.^62^ At high doses, the cell is forced to evaluate whether it can rescue itself by initiating metabolic processes that attempt to reverse or repair the biomolecules that have been oxidatively damaged.^85^ Irreversibly damaged cells are marked for destruction.

Since H_2_O_2_ is a long-lived ROS and considered one of the stable plasma-generated RONS, we will use that as an example to track the fate of plasma RONS. Inside the cell, H_2_O_2_ is a vital molecule important for several physiological functions, including proliferation, differentiation, and mediating immune responses.^86^ These effects are concentration, cell, and location dependent and mediated through oxidation of thiol residues. Cancer cells usually maintain a higher H_2_O_2_ level, thus enabling their unchecked growth.^87^ But higher levels of H_2_O_2_ may cause cell cycle arrest and cell death. To achieve the desired amount of H_2_O_2_ rapidly, the cell employs detoxifying enzymes like glutathione peroxidases, catalases, and peroxiredoxins. When the oxidants exceed the capacity of these enzymes, the cell initiates the oxidative stress responses, upregulating other pathways to breakdown H_2_O_2_.^86^ The dysregulation of these paths contributes to the development of cancer by allowing these cells to gradually adapt to the higher basal redox and enabling pathways that promote proliferation and angiogenesis.^86^ If this balance is further overwhelmed by exogenous sources (radiation, chemotherapy, etc.) apoptotic pathways (immunogenic and non-immunogenic) are upregulated.

Peroxynitrite is a short-lived specie (half-life 10–20 ms) produced by the cell and is important for many immune functions.^88^ At high doses, it is quite toxic and is used by phagocytic cells to destroy invading pathogens. It can also be toxic to mammalian cells, causing oxidative damage up to two cell diameters away^89^ via oxidative damage to DNA, proteins, and lipids.^88^ GSH serves as an efficient scavenger of peroxynitrite, and it is shown to react with GSH more readily than it is broken down.^72^ Plasma-generated ONOO can diffuse into the cell through the cell membrane, and its effects are concentration dependent. Cellular enzymes can generate more superoxide from peroxynitrite, providing a secondary mechanism of potentiating plasma-mediated cytotoxicity. In addition, through its nitrosylation activity, peroxynitrite changes the protein structure and function, thus affecting cell fate.

The interactions between RONS and the antioxidant system in cells serve as the molecular interface that helps to modulate metabolic and environmental signals to maintain cell and tissue health. It can, therefore, be expected that plasma may be used strategically to modulate the balance between RONS and cellular antioxidants in a feedback mechanism for its biological effects. Here, better understanding of key effectors for each application is needed.

### C. RONS and cell–cell communication

Cells communicate with each other, either directly through gap junctions and surface proteins or through chemical mediators.^90^ These are important for survival of the organism because it allows them to respond to changes in local and global environment. It also allows cells to communicate stress to their neighbors, asking for help and coordinating adaptive responses. RONS are proposed to facilitate such communication, either through direct transport between neighboring cells through bidirectional channels or through indirect signaling pathways like those that involve calcium ions.^91^ Promoting the secondary release of stress molecules like ATP, plasma RONS modulate immune responses in organisms.^92^ Changes in membrane protein composition also promote engagement of innate immune responses.^93–95^ These effects of plasma RONS are being explored for the development of plasma-based immunotherapy for cancers and some infectious diseases.^96,97^ The key is controlled delivery to induce only the desired effects.

Since each plasma device produces a unique cocktail of effectors, chemical and physical, research is needed to optimize this controlled delivery for each device. Furthermore, the dose-dependent response of cells must be considered for developing treatment regimens for different diseases in different organ systems.

## V. CELL RESPONSE TO ELECTRIC AND ELECTROMAGNETIC FIELDS, ELECTRICAL FEEDBACK

The effects of electric and electromagnetic fields on cells have been extensively investigated in a wide range of frequencies with most research up to and in the radio frequency (RF) range and more recent studies extending in the microwave and higher ranges in relation to the safety of communication devices.^98–101^ The studies show an increase in ROS in response to exposure to electromagnetic fields in frequency ranges relevant to plasmas produced by kilohertz, radio frequency, and nanosecond pulsed power sources. A simple model was proposed to explain a wide range of biological activity of pulsed and variable electromagnetic fields.^102^ The model is based on induced ion oscillation at the surface of the cell membrane that can affect electro-sensitive channels and disrupt the cell's electrochemical balance and function. Reversible and irreversible electroporation or changes in membrane permeability have been used for enhanced drug delivery and transfection. The same effects can also accompany plasma treatment of cells.^103^ Recently published results indicate that the application of a discharge generates electric potential in the solution covering a cell culture.^104,105^ Cells exposed to this potential stimulation without any exposure to plasma-generated species showed an increased membrane permeability to large molecules (temporary electroporation) with low lethal effects. This suggests that the plasma-induced potential in the liquid may enhance the action of plasma-generated RONS. Here, it is vital to note that synergism with the biological effects of plasma RONS on the molecular components of the cell membrane may also play an important role.^106–108^ The work of Schoenbach *et al*. showed that high intensity electric fields with pulse durations shorter than membrane charging time penetrate the cell and affect the cell organelles, damage DNA, and cause apoptosis.^109^ Their original hypothesis was based on a simple circuit model where the role of cell and intracellular membranes was represented by capacitors and medium, cytoplasm, and nucleoplasm as resistors. The circuit analysis provided an estimate and understanding of the frequency dependence of the cell response to external electromagnetic fields. It is well known that pulses of millisecond to microsecond duration can cause electroporation of the cell membrane but do not affect cell organelles, but nanosecond pulses do penetrate the cell and can cause DNA damage and apoptosis. More information on this vast field can be found in Refs. 110–112. The electrical nature of cells and their response to electromagnetic fields during direct plasma treatment can directly affect *in vitro* and *in vivo* plasma treatment.

### A. Electrical feedback effects

In addition to chemical feedback and chemical interactions between the plasma and the treated biological target, direct plasma sources in contact with the biological target also couple electrically, providing another path for feedback and interactions. In this section, we examine the device generating plasma with the liquid containing organic matter as a part of the overall electrical circuit.

The feedback effects from plasma–wall (such as the walls of a fusion or a plasma processing reactor) and plasma–target (such as in surface modification or medical applications) interactions have been studied for decades in fusion and low-temperature plasma for surface modification including the effects of sheaths and sputtered or evaporated impurities. Recently, the feedback effects relevant to biological applications have begun to come into focus. It was observed that placing a grounded plate beneath a Petri dish or a well plate containing the liquid cell sample changed the properties of plasmas.^113–118^ Physical properties of the sample, such as conductivity, permittivity, surface roughness, and surface energy, set up the boundary conditions for the distribution of fields, determining sheath formation. This, in turn, impacts discharge initiation and propagation and, hence, the energy in the plasma and fluxes of ions, electrons, and reactive species to the surface. Fluxes to the target lead to surface charging and modification of the chemical and physical properties of the sample. As the properties of the sample provide the boundary conditions for the plasma, this creates a feedback loop to the plasma.^7,119,120^

Surfaces in contact with plasma are exposed to plasma-generated reactive species, ions and electrons, and UV radiation, resulting in surface charging, photoionization, secondary electron emission, de-excitation, radical reactions at the surfaces, etc. The interactions between plasma and the target can be characterized based on the target conductivity, dielectric permittivity, and floating or grounded circuit connection. A metal substrate increases the radical production in a plasma jet hitting the surface,^121^ and propagation of the ionization wave to the surface, secondary discharge formation, and discharge propagation along the surface are all affected by substrate properties.^122^ A detailed discussion of the properties of plasma jets and their interactions with surfaces can be found in Ref. 119. As discussed above, the time scale of charge relaxation as compared to the plasma characteristic time affects charge accumulation and, hence, streamer formation along the surface. Liquids with conductivities below ∼10 *μ*S cm^−1^ behave as dielectrics for applied voltage pulse durations of ∼100 ns, but materials with conductivities on the order of 1 S cm^−1^, such as cell culture solutions, dissipate charge on a sub-nanosecond time scale.^120^

Due to the higher mobility of electrons in the plasma, dielectric surfaces are negatively charged. Ions accelerated to the surface are neutralized at the surface and produce secondary electron emission. The neutral species can then return to the gas phase. There may be other reactions at the surface, including but not limited to photoionization, de-excitation, and radical reactions at the surface. The net charge at the surface depends on the dielectric constant of the material. Simulations showed that electric fields inside high dielectric constant materials (>4) develop mostly due to surface charging, while volume charges may contribute to the field inside materials with lower dielectric constant.^123^

The relatively higher electric field in the gap for substrates with a higher dielectric constant can also be explained from the perspective of circuit impedance. Higher dielectric constant of a material leads to higher capacitance and lower impedance of the target material. This results in a lower voltage drop across the material and a higher voltage drop and E field in the gap for the same voltage pulse applied to the high voltage electrode. Therefore, a change in the dielectric properties of the target material influences the discharge inception, propagation, and plasma properties. Higher dielectric constant also leads to greater charge accumulation and a more intense return stroke observed in AC discharges during the opposite half-cycle and in pulsed discharges during the fall stage of the voltage pulse.^114,119,123^ Microscopic modeling approaches are getting better and better at predicting the interaction in the plasma channel, at the surface, and even into the top few layers at the surface, but they cannot address the changes in the entire system. Circuit models may be employed to provide insight into the interactions from the electrical point of view and address the issue of reproducibility and stability of the discharge conditions.^124^

**FIG. 5.**
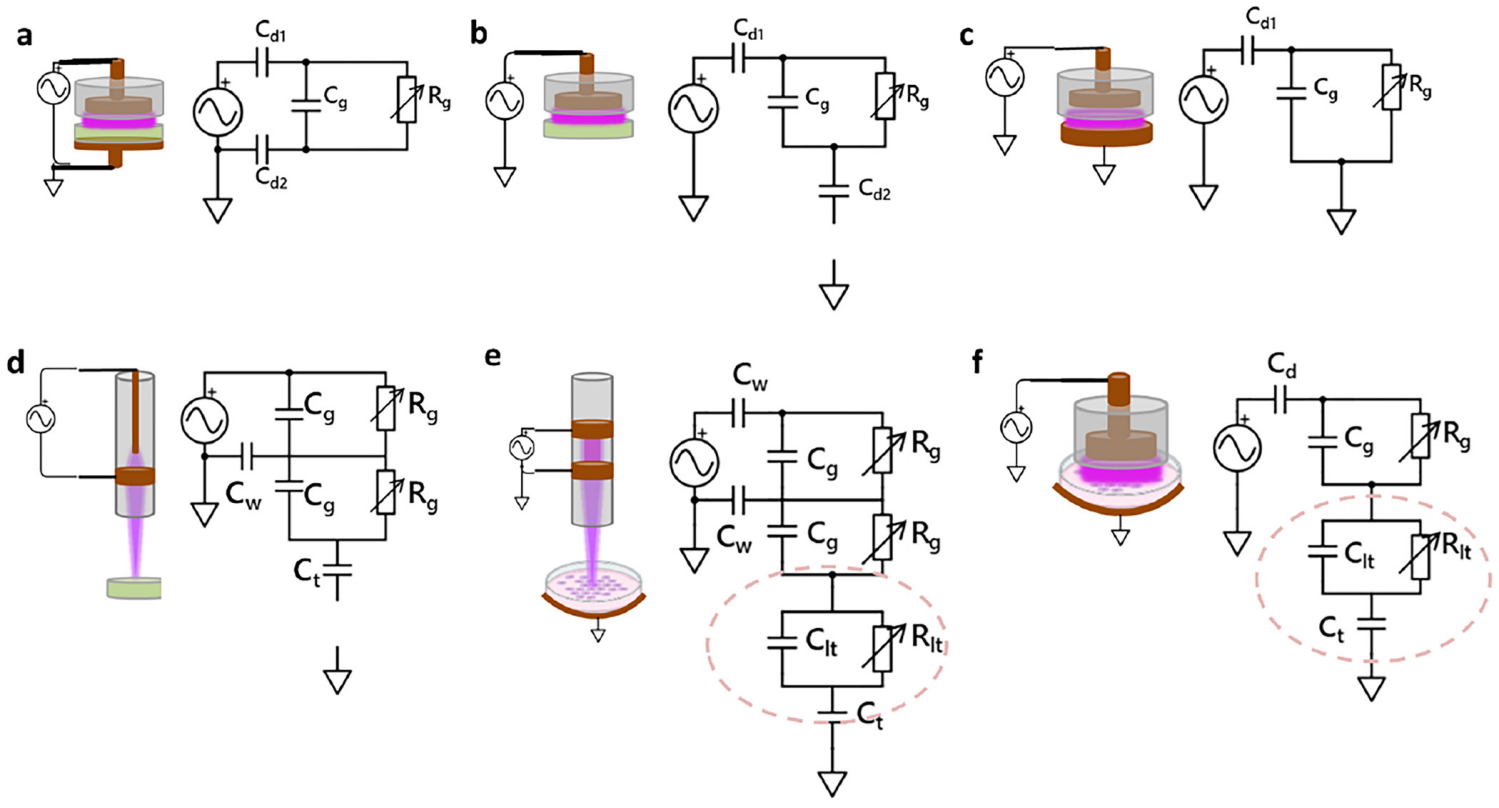
Equivalent circuit approach for volume DBD (a)–(c) and DBD jet systems (d) and (e). The biological target becomes part of the electric circuit (e) and (f), shown as the dashed ovals.

An equivalent circuit model approach considers the electrical properties of the entire system. Several typical DBD plasma systems and their respective equivalent circuits are shown in Fig. 5. In the case of a double dielectric barrier parallel electrode DBD (both electrodes are covered with dielectric material represented as d1 and d2 in Figs. 5(a)–5(c), the circuit may be represented as three capacitors connected in series, one corresponding to the air gap $\left(C_{g}\right)$ and two corresponding to the dielectric material covering the electrode, $C_{d1}$ and $C_{d2}$.^125^ Upon ignition, the air gap is bridged by plasma and may be considered a resistor $\left(R_{g}\right)$. This approach clearly shows the differences in the electric circuit when the substrate is grounded and composed of metal (5c) or covered with a dielectric (5a), or a floating dielectric (5b). A similar approach may be applied to study plasma jets. Two different jet configurations are shown in Figs. 5(d) and 5(e), where plasma is generated inside a dielectric tube and protrudes to the treated substrate. The capacitances due to the dielectric are marked $C_{w}$, where w is the wall dielectric material. As in the case of parallel electrodes, a variety of circuit configurations are possible resulting in different power output from the source due to the corresponding load impedance in each situation.

In medical and biological applications, the biological target becomes part of the circuit [Figs. 5(e) and 5(f)]. The dashed ovals indicate the liquid with organic matter *in vitro* in a grounded configuration. The liquid/cell target is treated as a leaky capacitor with a capacitance $C_{\mathrm{lt}}$ and resistance $R_{\mathrm{lt}}$, and the capacitance of the dielectric dish is $C_{t}$. The equivalent circuit approach shows that changes in dielectric properties and conductivity of a liquid target will affect the circuit impedance. These changes can occur during plasma exposure of the target transiently. The systems of liquid/cells in a Petri dish are simple, but *in vivo* applications include an entire body surrounding the treated area. Stancampiano *et al*. show that the same plasma source has different electrical characteristics when applied to tissue or cells *in vitro*, compared to a mouse and a human.^126^ The changes in properties of liquid/cell targets will affect the circuit impedance and, hence, the power delivered to the target, setting up an electrical feedback loop. Additional investigations are needed and strongly encouraged to address the translation of a plasma system from one application to another. Based on these, we conclude that electrical characterization of plasma devices should be conducted for each specific application, and real-time electrical monitoring is essential during plasma treatment.

A recent study investigated the electrical feedback loop using a floating electrode DBD driven by a kHz AC power source and cells in suspension.^5^ In this configuration [Fig. 5(f)], the effective circuit impedance during the discharge phase can be approximated as

$$Z_{\mathrm{eff}}=\sqrt{R_{g}^{2}+{\left(\frac{1}{2\pi fC_{\mathrm{eq}}}\right)}^{2}},\;\mathrm{with}\;C_{\mathrm{eq}}=\frac{C_{lt}C_{d}C_{t}}{C_{lt}C_{t}+C_{d}C_{t}+C_{d}C_{lt}},$$

and $C_{\mathrm{clt}}$, $C_{d}$, and $C_{d2t}$ are the capacitances of the liquid with cells, the dielectric covering the top electrode, and the dielectric covering the electrode underneath the cell culture (a Petri dish, glass cover slip, or another cell culture support). The effect of $C_{\mathrm{eq}}$ on the overall impedance depends on the geometry of this layer; for layers of very high capacitance, the effect may be less significant than if the layer capacitance is on the same order as $C_{d,t}$. The equivalent circuits in Figs. 5(e) and 5(f) indicate that a similar argument applies to the jet systems; the biological target is part of the electrical system as seen by the driving power source. It should be noted that this applies to direct contact plasma sources, the focus of this Perspective.

Cells are enclosed by a lipid membrane and are filled with electrically charged ions that confer biophysical properties like membrane capacitance and cytoplasm conductivity^127–129^ They maintain an electrical potential difference across the membrane by actively pumping these charged ions. The membrane plays a key role in the dielectric properties of cells, in particular, their dielectric permittivity and its dispersion relation, properties that are used for cell identification, for measuring cell proliferation and migration, and for assessing cell viability.^130–133^ Since the liquid cell culture medium containing cells is part of the electric circuit, any changes during plasma exposure are likely to impact the whole system. The methods for measuring permittivity of cells and cell suspensions rely on measuring the complex impedance of cells culture alone or in combination with a substrate using specially designed circuits using lock-in amplifiers or Vector Network Analyzers (VNAs). Known as dielectric spectroscopy, these methods of measuring cell permittivity have become a standard addition to electrokinetic methods such as dielectrophoresis and electrorotation.^131,133–135^ Experiments using dielectric spectroscopy have also been performed to examine the correlation between plasma properties and the capacitive signatures of the cell cultures.^124,136^

The variations in cell dielectric properties, their effect on the properties of cell cultures and suspensions, and the observation that plasma properties are sensitive to the capacitance of cell cultures suggest that in some configurations, cell dielectric properties can influence the global properties of the plasma-generating circuits. We tested this hypothesis using Vero cell suspensions in cell medium that contained live cells or “killed” cells (heat treated, cells lysed by repeated freezing and heating, and cells fixed with paraformaldehyde).^5^ The cells were subjected to non-damaging, short (20 s) duration pulsed plasma treatment with a floating DBD system, and Lissajous figures plotted from the electrical characteristics were used to assess the changes in the circuit impedance and the discharge energy per cycle.

**FIG. 6.**
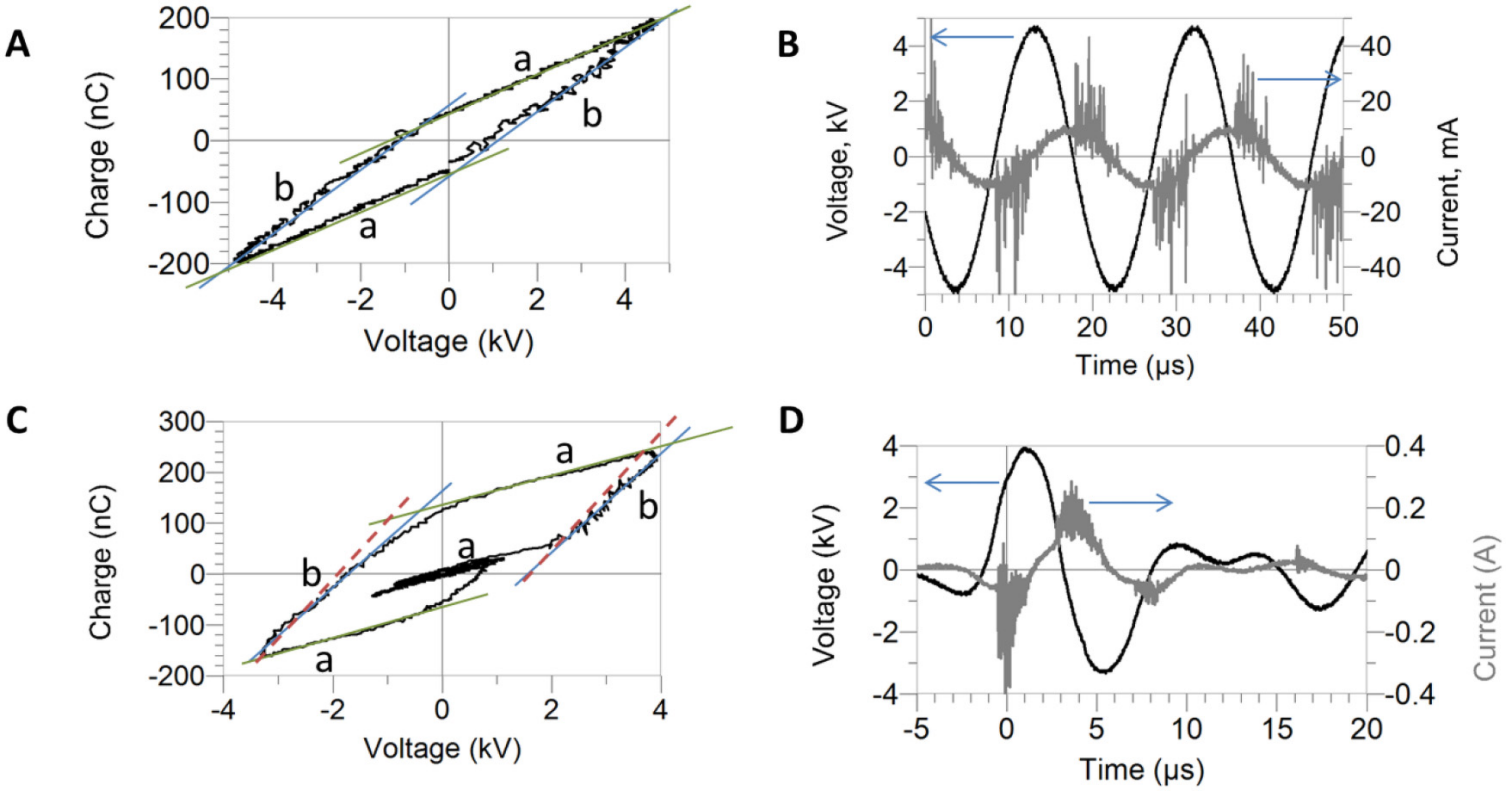
Lissajous figures (a) and (c) and measured voltage and current (b) and (d) for AC (a) and (b) and pulsed (c) and (d) DBDs.

Electrical characterization of DBD systems usually includes the measurements of the voltage between the high voltage electrode and the ground, the current to ground, and the charge transferred [Figs. 6(b) and 6(d)]. The Lissajous figure, a plot of the charge transferred vs the applied voltage, may be used to gather characteristics of the circuit. The classical ideal shape of a Lissajous figure for a DBD is a parallelogram [see Fig. 6(a)], comprised of lines **a**, corresponding to the part of the applied voltage cycle without any discharges and sides **b**, corresponding to the microdischarges seen as current spikes [Fig. 6(b)]. A variety of shapes are possible determined by the development of the discharge and the changes in the properties of the target.^137–139^ The slope of the Lissajous figure dQ/dt during the discharge portion [sides **b** in Figs. 6(a) and 6(c)] of the power cycle can provide insight into the behavior of the dielectric materials including the cell tissue targets, as determined by the capacitive portion $Z_{\mathrm{eff}}=\frac{1}{2\pi fC_{\mathrm{eq}}}$ ( $C_{\mathrm{eq}}$ defined above). The slope can be affected by the discharge spreading on the surface of the treated material (change in discharge area) and the properties of the material, which may also vary as a function of the applied electric field.^137,140–144^ As the shape of the applied voltage signal depends on the impedance of the system load, comprised of the plasma and the treated target, there is an interaction and a possible feedback loop between the changing properties of cells, affecting the overall impedance of the circuit, and the resulting plasma properties, determined by the applied electric field and changing boundary conditions.

Since a pulsed power source was used in our experiments,^5^ the Lissajous figures are slightly different but still contain the “no discharge” and “discharge” sections **a** and **b** [Figs. 6(c) and 6(d)]. The slope of the straight sections depends on the capacitance of the system, which for section **b** is influenced by the cell culture and the supporting dish or slide [cf. Fig. 5(f)]. A change in the cell condition resulted in the change in the slope of sections **b** as shown by the dashed lines in Fig. 6(c). These measurements based on the slope of the discharge section of the Lissajous figure showed a small but significant variation in the impedance between live and heat treated and between live and lysed cells (p < 0.005 by ordinary one-way ANOVA). Consequently, a difference in the deposited energy determined from the area of the Lissajous figures was also observed. Furthermore, when live cells were exposed to damaging conditions, measurements at the end of treatment also indicated an increase in the circuit capacitance and the energy per cycle delivered to the cells. This simple study indicates that cell cultures have an effect on the overall performance of the plasma driving circuit. Changes in power delivered to the liquid and cells are also likely to change the concentrations of reactive species as well as the biological outcomes. These results reiterate the importance of monitoring not only the end results but also *in situ* variations in the target conditions as well as the electrical characteristics of the power source. It is important to note that these changes can occur during treatment in a transient manner as the cell viability and morphology begin to change, which can only be discovered and monitored with *in situ* and real-time measurements.

The challenge of *in situ* and real-time measurements of *in vitro* and *in vivo* systems highlight the importance of measurements that do not interfere with the treatment parameters, such as optical emission spectroscopy, dielectric spectroscopy of the target, and electrical monitoring of the overall treatment system. A deeper understanding of the issues and relationships presented here requires continued multidisciplinary and multifaceted approaches for developing real-time, *in situ* characterization techniques as well as increasing our theoretical understanding of cellular and plasma responses and interactions. This is complicated by practical difficulties of measuring plasma composition and plasma parameters during treatment *in vitro* and *in vivo* without interfering with the treatment. For example, powerful laser measurement techniques cannot be applied during cell treatment, and the methods are limited to optical emission and fluorescence spectroscopy, dielectric spectroscopy, and electrical measurements. In the absence of one-to-one correspondence in these multiparametric systems and cause-effect relationships, machine learning (ML) techniques are being employed to control outcomes in biological and medical applications of plasma.^124,136^ Ml approaches including Physics Informed Neural Networks have shown some promise as demonstrated by the controlled selectivity in plasma exposure of cancer cells.^145^

## VI. SUMMARY AND OUTLOOK

In this Perspective, we explored the truly interactive behavior between plasma and biological systems occurring simultaneously at two interconnected levels: chemically and electrically. We advocated for the analysis of the system as a whole—plasma source, gas phase plasma, liquid phase, and the organic matter—and to raise the level of awareness regarding the importance of this complex system that interacts at multiple levels. Detailed knowledge of the transport behavior of RONS from the gas phase into the liquid phase (Sec. II) is crucial to understand the complex processes and interactions when organic matter is introduced in the liquid (Sec. III). We argued why the system needs to be studied as “one system” and motivate more research toward transport processes and interface chemistry. Plasma–organic matter interactions are dynamic and co-evolve, in particular when living organisms are present (Secs. IV and V). Some changes occur in real time and others develop slowly over minutes, hours, or even days later. Static measurements do not reflect the true picture, raising questions and challenges for future research: How does the cell response contribute to plasma–liquid chemistry? And how does changing chemistry feed back to the plasma discharge? More research and development efforts need to be encouraged that will investigate measurement techniques that allow for real-time measurements with high spatial resolution, capable of resolving the plasma–liquid interface either directly or indirectly. It is obvious that this level of analysis requires close collaborative efforts among scientists in diverse fields. Funding agencies should be encouraged to support such cross-disciplinary research, for example, by expanding inter-agency funding announcements or by continuing and expanding support for User Facilities that are able to provide *in situ* and time-resolved plasma diagnostics and expertise. Technically, measurements of physical and chemical changes must be made in the presence of the biological target, ideally during exposure to plasma. In addition, the evolving chemical changes because of ongoing reactions among plasma species and those produced by the cells must also be investigated. Biological changes should be similarly characterized kinetically starting immediately after exposure to plasma and spanning several hours to days, depending on the intended outcome. Availability of *in situ* live cell imaging technology makes this feasible.

In addition to the chemical feedback and coupling, there is electric coupling and potential feedback from the living target back to the plasma. Physical properties such as electric fields and surface charging affect cell properties, dielectric properties of the target material, and the transport across the interface. We advocate for monitoring the electrical behavior of the complex system as one system *in situ* and to monitor changes in dielectric properties of materials and changes in the gap electric field during plasma treatment of targets. The addition of ML/AI approaches can fill the gap in available data through the application of physics-based neural networks and may help to adapt the treatments to variations in the system, including source and target.^146^ Since all these components of the system affect the plasma discharge, the therapeutic outcome will depend on the dynamic interactions and modifications of each component.

## Data Availability

Data sharing is not applicable to this article as no new data were created or analyzed in this study.
